# Iron in Multiple Sclerosis and Its Noninvasive Imaging with Quantitative Susceptibility Mapping

**DOI:** 10.3390/ijms17010100

**Published:** 2016-01-14

**Authors:** Carsten Stüber, David Pitt, Yi Wang

**Affiliations:** 1 Department of Radiology, Weill Cornell Medical College, New York, NY 10044, USA; yw233@cornell.edu; 2 Department of Neurology, Yale School of Medicine, Yale University, New Haven, CT 06511, USA; david.pitt@yale.edu; 3 Department of Biomedical Engineering, Cornell University, Ithaca, NY 14853, USA

**Keywords:** MS lesion, quantitative susceptibility mapping (QSM), deep grey matter (DGM), MRI

## Abstract

Iron is considered to play a key role in the development and progression of Multiple Sclerosis (MS). In particular, iron that accumulates in myeloid cells after the blood-brain barrier (BBB) seals may contribute to chronic inflammation, oxidative stress and eventually neurodegeneration. Magnetic resonance imaging (MRI) is a well-established tool for the non-invasive study of MS. In recent years, an advanced MRI method, quantitative susceptibility mapping (QSM), has made it possible to study brain iron through *in vivo* imaging. Moreover, immunohistochemical investigations have helped defining the lesional and cellular distribution of iron in MS brain tissue. Imaging studies in MS patients and of brain tissue combined with histological studies have provided important insights into the role of iron in inflammation and neurodegeneration in MS.

## 1. Introduction

Iron as an electron facilitator is involved in healthy brain functions including myelin production, oxygen transport and neurotransmitter synthesis [1,2,3]. Excessive brain iron levels are invariably associated with neurodegenerative diseases, such as Alzheimer’s disease, amyotrophic lateral sclerosis and Parkinson’s disease [1,4]. Multiple sclerosis (MS) is an inflammatory disease of the central nervous system that also has neurodegenerative features [5,6]. Iron has been shown to accumulate in deep grey matter (DGM) nuclei of patients with MS and in white matter (WM) lesions [7,8,9,10]. Thus, there is an increased interest in quantifying iron in MS patients with noninvasive magnetic resonance imaging (MRI) that is sensitive to the magnetic field generated by tissue iron [8,9,10,11,12,13,14,15,16,17,18,19,20,21,22,23,24,25,26,27,28,29,30,31,32,33,34,35,36,37,38,39,40,41,42,43]. However, conventional MRI methods for detecting or measuring iron suffer from blooming artifacts due to the nonlocal nature of magnetic field. Quantitative susceptibility mapping (QSM) [44,45] provides a field-to-source deconvolution approach to iron quantification, overcoming the blooming artefact problem in conventional MRI [41,46]. Here, we review the physiological role of iron in the healthy brain, the progress made in brain iron mapping through MRI methods and the contribution of iron to MS pathophysiology.

## 2. Iron Detection Methods

We briefly review the tools that can be used to detect iron in tissue, including histological techniques and elemental analysis tools to quantitatively measure iron in tissue samples. We also review non-invasive MRI techniques for *in vivo* study of brain iron, which are important for translating tissue studies into clinical practice and for *in vivo* biological and pathophysiological investigations.

### 2.1. Ex-Vivo Iron Detection and Measurement

We first discuss qualitative and quantitative *ex vivo* methods for studying tissue iron, which can be considered as the gold standard for studying iron chemistry and quantity.

#### 2.1.1. Qualitative Detection of Iron—Histology

Fe^3+^ staining (Perls’ Prussian blue) and Fe^2+^ staining (Turnbull’s blue) have been used for qualitatively exploring the distribution of iron in brain tissue. Because most iron in the brain is present as ferric (Fe^3+^) iron, Perls’ staining is the most commonly used technique to visualize iron histologically. In addition, immunohistochemical labeling is used for iron-related proteins, including ferritin [10,47,48], transferrin [47,49,50] and ceruloplasmin [51]. However, presence of ferritin does not necessarily reflect presence of iron, as has been shown in myeloid cells where ferritin can be present in the absence of iron [9]. 

#### 2.1.2. Quantitative Detection of Iron—Spectrometric Techniques

A few spectrometric techniques are available to quantify iron in biological tissue, which are either based on the characteristic atomic mass [52] or element-specific characteristic X-rays emitted from atomic inner shell transitions [53]. Spectrometric methods measure iron irrespective of its chemical state, *i.e.*, Fe^2+^ or Fe^3+^, thus quantifying the total iron content. Here is a brief outline of commonly used iron quantification methods:

- **Colorimetry**: This iron-detecting approach requires a standard of the measured absorbance for known concentrations, thus making it problematic for comparing tissue samples of different measurements [47,54,55].
- **Atomic Absorption Spectrometry** **or** **Spectrophotometry (AAS)**: The technique equally requires standards with known analyte content to establish a relationship between measured absorbance and analyte concentration [56,57,58].
- **Instrumental Neutron Activation Analysis (INAA)**: The intensity and wavelength of the signal is element-specific and can be used to characterize the examined sample [59,60,61].
- **Inductively Coupled Plasma Mass Spectrometry (ICP-MS)** [4,62,63]: ICP-MS has become the most prominent method to quantitatively correlate iron concentration and MR-signal, in particular iron and R2* [17,64,65,66,67], although a quantitative mapping of the elemental distribution is not possible. However, ICP-MS in combination with laser ablation (LA-ICP-MS) allows for microlocal element analysis [68,69]. LA-ICP-MS has been use to quantitatively map the iron distribution of brain tissue [41,70].
- **X-ray Fluorescence Spectrometry (XRF)** or Rapid Scan X-ray fluorescence spectrometry (RS-XRF) [71,72]: This technique allows for iron mapping and experiments have been conducted at two different locations; at the Stanford Synchrotron radiation laboratories (SSRL) [73] or more recently at the new Diamond light source in Oxfordshire, UK [74].
- **Proton-Induced X-ray Emission (PIXE)** [75,76,77]: A similar principle as XRF allowing to create iron maps of brain tissue [78,79,80].

RS-XRF, PIXE and LA-ICP-MS are the only techniques that can been applied to correlate MR signal and local iron concentration voxel by voxel [41,71,81]. These techniques can be utilized as the gold standard for mapping the local iron tissue concentration.

### 2.2. In Vivo Iron Detection and Measurement

Noninvasive MRIs are highly sensitive to tissue iron content and has become the method of choice for investigating brain iron, including iron in MS brains. We review first the qualitative MRI methods for detecting iron, semi quantitative methods for measuring iron, and then describe quantitative susceptibility mapping (QSM) for biophysics model based iron quantification.

#### 2.2.1. Qualitative MRI Methods

MRI offers rich tissue contrasts to sensitize tissue compositions and biophysical processes. Traditional T2 and T2* relaxation contrasts have been used to detect brain iron; T1 relaxation has been shown to be slightly affected by iron, but not as strongly as T2 or T2* [81,82,83,84,85]. Here, we summarize T2 and T2* based methods for detecting brain iron:

- **T2-weighted imaging (T2w).** Iron appears hypointense on T2-weighted images because paramagnetic iron produces a field in its surroundings, contributing to the incoherent thermal dephasing, *i.e.*, enhancing the transverse relaxation rate 1/T2 = R2. Iron R2 enhancement increases with magnetic field strength [12]. Iron concentration has been shown to linearly correlate with R2 in DGM nuclei where iron distribution may be regarded as uniform [12,82,86,87,88]. However, the relationship remains difficult in both cortical grey matter (GM) and WM, due to the complex geometry of iron distribution [87].
- T2* weighted imaging (T2*w). This magnitude gradient echo imaging (GRE) based T2* contrast is sensitive to the intravoxel variation of the iron induced magnetic field [89], in additional to the T2 contrast. T2*w is more sensitive to iron than T2w and has become the method of choice for *in vivo* iron detection [90,91,92]. An enhanced version of T2*w is susceptibility weighted imaging (SWI), which applies a phase attenuation to further increase the hypointensity contrast in T2*w [17,93]. SWI contains more blooming artifacts than T2*w. However, T2*w and SWI do not directly reflect the iron concentration due to their blooming artifacts and dependence on imaging parameters.

#### 2.2.2. Semi-Quantitative MRI Methods

Quantifying iron from MRI data has long been a desired goal. A number of MRI metrics have been investigated, which are proportionate to iron content in limited situations. However, these methods are essentially based on the magnetic field, which contains blooming artifacts, *i.e.*, depends on both local iron in a voxel and iron outside the voxel of interest.

- **Magnetic Field Correlation Imaging (MFC)** is directly related to iron-induced field variations and has been used to estimate iron concentration [22]. MFC bears similarity to R2’ that is determined by the field variance in a voxel. MFC is estimated from a number of asymmetric spin echoes that are similar to gradient echoes [94,95].
- **Phase Imaging.** The gradient echo MRI data contains both magnitude and phase images. Traditionally, the phase images have been discarded and only the magnitude images are saved on the MRI scanner. The phase shift divided by the gyromagnetic ratio is the echo time times the iron induced magnetic field, which is proportional to the iron concentration convolved with the dipole kernel [96,97].
- R2* mapping. A number of correlation studies show that iron content is linearly related to the 1/T2* = R2* in DGM nuclei where iron distribution is approximately constant [87,98]. However, R2*, consisting of iron-enhanced R2 and iron-caused intravoxel dephasing (R2’), does not reflect local tissue iron concentration when its distribution is nonuniform. As a consequence, solid and shell MS lesions might be indistinguishable on R2* maps. Furthermore, R2* depends on imaging parameters [66,99], and suffers from blooming artifacts, nonlinearity, background field variations and other errors [99,100,101,102,103,104].

In summary, current traditional MRI quantification methods do not provide absolute quantification (some scaling factor remains to be determined), and suffer from being non-local, *i.e.*, their values at a given voxel depend not only on the iron content in that voxel but also on iron concentration in the nearby voxels with a distance and direction dependent weighting [105].

### 2.3. Quantitative Susceptibility Mapping (QSM)

The GRE phase images or, more precisely, the magnetic field can be deconvolved to accurately determine the distribution of magnetic source—tissue susceptibility, which is quantitative susceptibility mapping (QSM). QSM eliminates blooming artifacts and quantifies the local tissue magnetic properties [44,106,107]. This field to source inversion has been made possible through the use of Bayesian inference and anatomic prior knowledge [44,45]. The inversion from field to susceptibility is known to be ill-posed [108]: there are zeroes in the kernel connecting the susceptibility distribution and the field, and a simple kernel division causes large errors that are present as streaking artifacts in the reconstructed susceptibility map [109,110]. Regularization is necessary to select a unique susceptibility solution for a given field [110,111,112,113,114]. In recent years, various regularizations have been tried for QSM reconstruction [44,106,107,115,116,117,118,119,120,121,122,123,124,125], reflecting different ways to impose known anatomic structural information in the desired QSM; they all demonstrated similar results [45]. While the QSM technology is still being actively developed, particularly to account for myelin contribution to magnetic susceptibility, current QSM is sufficiently robust and reliable for mapping highly paramagnetic brain iron, the dominant contributor to tissue susceptibility [110,126,127]. QSM divided by iron molar susceptibility generates the iron mass map for the iron-rich DGM nuclei [98,115].

## 3. Iron in Healthy Brains

Iron is the most abundant trace metal in the healthy human brain, at levels 20–30 times higher than all other trace metals combined [128,129]. Iron levels increase nearly linearly with age from 0–20 years old for a developing brain, then they stay almost constant until 60 years old for a mature brain, and then further increase with age, ultimately causing neurodegeneration [54].

The majority of brain iron is present in the inactive form of ferric iron (Fe^3+^) stored in the spherical shell of protein ferritin [130]. A small amount (~5%) of brain iron is present in the active form of ferrous iron (Fe^2+^). Iron transporter proteins including transferrin, DMT1 (divalent metal transporter) and ferroportin can transport Fe^2+^ ions. When ferrous iron is not needed, the H-subunit of ferritin oxidizes ferrous iron into ferric iron and stores it in ferritin [49]. On the contrary, ferric iron can be readily converted by reductants and chelators into ferrous iron [131]. The brain iron level is delicately maintained by iron-regulating proteins for ferritin, iron transporters, and transporter receptors, forming a precise iron homeostasis that is critical for healthy brain function [132]. Disruption of brain iron homeostasis leads to neurological diseases, where iron overloading in various part of the brain leads to neurodegenerative diseases, including MS [1,9,10,36].

Brain iron is stored in tissue cells where neurochemical processes require iron. The highest iron concentrations are found in the nuclei in the DGM in the midbrain, including globus pallidus (21 ± 3 mg iron/100 g fresh weight (fw), mature brain), red nucleus (19 ± 7 mg iron/100 g fw), substantia nigra (18 ± 7 mg iron/100 g fw) and putamen (13 ± 3 mg iron/100 g fw) [54]. These midbrain nuclei constantly generate neurotransmitters including dopamine and glutamate to maintain signal traffic in brain circuits, and these neurotransmitter syntheses seem to require electron facilitation by iron. There is also substantial amount of iron in cortical grey regions including the motor (5 ± 1 mg iron/100 g fw, mature brain), occipital (4.6 ± 0.7 mg iron/100 g fw), sensory (4.3 ± 0.6 mg iron/100 g fw) parietal (3.8 ± 0.7 mg iron/100 g fw), temporal (3.1 ± 0.6 mg iron/100 g fw) and prefrontal cortex (3 ± 0.4 mg iron/100 g fw) [54,109].

WM iron is more homogeneously distributed compared to the variations found in GM. The WM iron concentration is higher in the frontal lobe and lower in the occipital lobe compared to adjacent cortical GM indicating a gradient in iron concentration [48,81,133]. Cells containing iron cluster around blood vessels in WM give a patchy appearance on a microscopic scale [47,134]. Oligodendrocytes contain substantial amounts of iron [134], which is necessary for their large metabolic activity including the synthesis of myelin [60,62]. In the midbrain DGM, neurons and astrocytes also have a substantial amount of iron [51].

## 4. Iron in Multiple Sclerosis

The pathological hallmark of MS is inflammatory demyelination in both white and gray matter. Demyelination is mediated by myelin protein-specific T cells and myelin-phagocytosing macrophages. It is well documented that iron is retained by macrophages during inflammation [135]; however, macrophage iron uptake strongly depends on their state of activation. Activated macrophages can assume a continuum of polarization states, which may include one band of “classical” or proinflammatory M1 activation, and another band of anti-inflammatory M2 activation.

M1 macrophages secrete high levels of proinflammatory cytokines and reactive oxygen species and have increased microbiocidal capability while M2 macrophages dampen proinflammatory cytokine levels, have a high scavenging capacity and are essential for late phase tissue repair [136,137,138]. Moreover, M1 polarization is associated with high intracellular iron content, while M2 polarization is associated with enhanced iron release and low intracellular iron [139,140]. In addition, iron uptake itself has been shown to promote a proinflammatory state in macrophages through activation of NF-κB, a master regulator of the innate and adaptive immune system [141,142]. More recently, the M1-M2 paradigm for macrophages has been criticized as too bipolar and other models are now being proposed that take the complex and possibly mixed macrophage phenotypes into account [143].

### 4.1. Iron in White Matter MS Lesions

**Figure 1:**
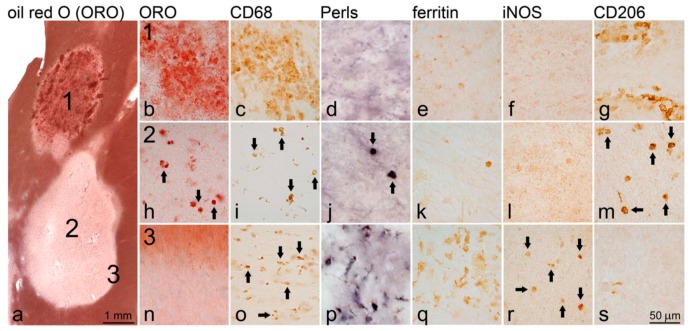
Iron deposition in white matter MS lesions. The overview image (**a**) shows an oil-red O-stained with an actively demyelinating lesion (**1**) and a demyelinated lesion (**2**); The actively demyelinating lesion [1] contains myelin-laden macrophages as indicated by oil-red O (ORO) positive material within CD68^+^ macrophages (**b**,**c**); These macrophages do not contain iron, indicated by Perls’ staining (**d**); or the iron-storage protein ferritin (**e**); and display markers indicative of M2 polarization (iNOS^−^ (**f**) and CD206^+^ (**g**)); The demyelinated lesion center (**2**) shows CD68^+^ macrophages (black arrows) containing condensed myelin (**h**,**i**); occasionally containing iron (**j**) and correspondingly ferritin (**k**); and expressing CD206 (**m**); but not iNOS (**l**). In contrast, the demyelinated lesion rim (**3**) shows microglia (black arrows) containing no ORO positive material (**n**,**o**); but large amounts of iron (**p**) and iron-storing ferritin (**q**);. These cells are iNOS positive (**r**) and CD206 negative (**s**) suggestive of M1-like polarization. (Source: [10]). The black arrows are indicating macrophages.

WM lesions are characterized by an initial infiltration of activated T cells and macrophages, demyelination and blood-brain barrier (BBB) breakdown. Iron is absent from early active, gadolinium-enhancing lesions as suggested by imaging studies of MS patients and by MRI and histological analysis of MS brain tissue [10,41,42,144]. At this early stage, macrophages contain myelin fragments and are M2 polarized [145], which is associated with low iron content (Figure 1).

A retrospective QSM imaging study of MS patients showed that lesion susceptibility values increase sharply as lesions become non-enhancing and remain high for several years [42]. Histologically, these lesions show no or minimal active demyelination but contain iron-rich microglia, predominantly at the lesion rim [146] (Figure 1). The presence of iron in these microglia cells presumably propagates chronic, low-grade and proinflammatory activation not associated with demyelination that may ultimately contribute to neurodegeneration and disease progression. The sources of iron in microglia in MS lesions are likely oligodendrocytes and myelin, who, upon their destruction, release iron into the extracellular space [9].

Finally, chronic silent lesions, *i.e.*, longstanding lesions that no longer contain inflammatory cells have low susceptibility values, thus indicating low iron content [42].

### 4.2. Iron in Cortical MS Lesions

Iron content has also been examined in cortical lesions. We found that activated microglia at the border of chronic active cortical lesions contained high amounts of iron [147], consistent with our findings in chronic active WM lesions. A study that examined silent cortical lesions, *i.e.*, lesions that did not contain inflammatory cells, found that iron density was reduced compared to normal appearing GM [148]. This iron loss is presumably caused by depletion of iron-containing oligodendrocytes and myelin sheaths from cortical lesions and possibly from neurons.

### 4.3. Iron in Normal Appearing White Matter (NAWM)

A recent histopathological report suggests that iron is lost from Normal Appearing White Matter (NAWM) and that this loss becomes more pronounced with increasing disease duration [9]. This observation is corroborated by an MRI study of MS patients that reports reduced R_2_’ signal in NAWM of MS patients as compared to controls [25]. Iron depletion from oligodendrocytes may compromise myelin and ultimately axonal integrity in myelinated white matter, a pathology that is thought to play a prominent role in chronic progressive MS [9,37].

### 4.4. Iron in Deep Gray Matter of MS Patients

**Figure 2:**
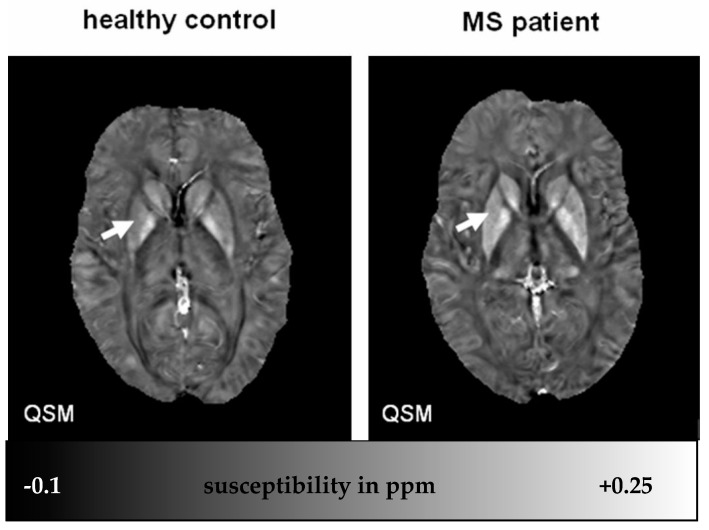
QSM map of a HC subject (29 y) and an MS patient (29 y). Note increased susceptibility in the basal ganglia of the MS patient indicating increased iron content. Susceptibility differences are most evident in the putamen (white arrow, 0.049 *vs*. 0.092 ppm). (Source: [46]).

Increased iron deposition has been observed in the deep nuclei of MS patients, predominantly in the putamen [33,34] (Figure 2). Moreover, it has been demonstrated that iron correlates better than any conventional measure with physical disability [149], brain atrophy [150,151,152], cognitive dysfunction [152] and risk of developing sustained progression of disability [88]. Iron accumulation in the basal ganglia during aging is accelerated in neurodegenerative diseases [2,153,154]. The cell type(s) in which iron accumulates in DGM in MS are currently unknown. Similarly, it has not been examined whether iron deposits are directly associated with cellular pathology. It is tempting to speculate that iron overload induces neuronal degeneration through enhanced free radical production and oxidative stress, as it has been shown in Parkinson’s disease [155].

In summary, brain iron homeostasis is profoundly perturbed in MS, involving different compartments and resulting in various pathologies. First, iron accumulates after initial acute demyelination in activated microglia at the rim of white matter lesions, where it might contribute to long-lasting (several years) neurotoxic inflammation. Moreover, with increasing disease duration, iron is slowly depleted from normal appearing white matter, which potentially compromises oligodendrocyte functions including myelin maintenance and contributes to axonal damage outside of white matter lesions [60]. Iron is also depleted in inactive cortical lesions, which may affect neuronal function. Finally, iron accumulates at an increased rate in the deep nuclei of MS patients. Although the pathogenic significance of this has not been elucidated, iron deposition has been shown to correlate well with various measures of disability in MS and to be associated with neurotoxicity in other neurodegenerative diseases.

## 5. Investigating Iron in MS

*Ex-vivo* and *in vivo* qualitative and quantitative detection and measurement of brain iron levels is very useful for understanding the role of iron in MS pathogenesis, and for translating *in vivo* MRI patient studies.

### 5.1. Ex-Vivo Methods to Detect Iron in MS

A number of studies have used histology to characterize iron content in post-mortem brain tissue of MS patients, either employing Fe^3+^ stain [31,41,147], in combination with immunohistochemical detection of ferritin [10,144], or Fe^2+^ stain [9,33,51] and in combination with ferritin [20,28]. Only one study has used a quantitative technique, LA-ICP-MS, to measure the iron concentration in MS lesions (Section 5.3.4; Figure 5) [41].

#### 5.1.1. Immunohistochemical (IHC) Analysis of Iron in MS Lesions [10]

We explored the role of lesional iron in multiple sclerosis using multiple approaches: immunohistochemical examination of autoptic MS tissue (Figure 1), iron-uptake in human cultured macrophages and MRI of relapsing and secondary progressive MS patients. Using Perls’ stain and immunohistochemistry, iron was detected in MS tissue sections predominantly in non-phagocytosing microglia at the edge of established lesions. Moreover, iron-containing microglia, but not myelin-laden macrophages, expressed markers of proinflammatory (M1) polarization. Similarly, in human macrophage cultures, iron was preferentially taken up by non-phagocytosing, M1-polarized macrophages and further induced M1 (super)polarization. Iron uptake was minimal in myelin-laden macrophages and active myelin phagocytosis by iron-laden macrophages led to the depletion of intracellular iron.

Iron deposits were more prevalent in WM lesions of patients with active relapsing-remitting MS than in patients with stable, longstanding disease. Together, these data suggest that iron accumulation in WM lesions are indicative of chronic, non-demyelinating inflammatory activity that occurs behind a closed blood-brain barrier and can be detected by QSM.

### 5.2. In-Vivo Methods to Detect Iron in MS

Noninvasive MRI has been the main window for studying MS. In particular, non-conventional MRI techniques have been increasingly used to better probe the MS pathological substrate, including Gradient echo (GRE) imaging, which is highly sensitive to iron and myelin distribution.

#### 5.2.1. Qualitative Iron Detection Using Conventional MRI Contrasts

The magnetic susceptibility weighting in T2* contrasts becomes more prominent in Ultra-High Field MRI, allowing the study of DGM iron deposits with increased sensitivity for basal ganglia regions [8,144,156,157] and WM lesions [158], and enabling investigation of small cortical lesions [159]. The noninvasive nature of MRI allows longitudinal studies to investigate the change of iron concentration over the progression of MS. Several longitudinal studies were performed in DGM structures and consistently showed a correlation between an increases in iron concentration with disease duration [38,39,40]. It has been found that iron accumulation is more pronounced in the early phase of the disease compared to the later stage [40]. The largest iron increase was observed in the substantia nigra and the globus pallidus in relapsing-remitting multiple sclerosis (RRMS) patients [38].

**Figure 3:**
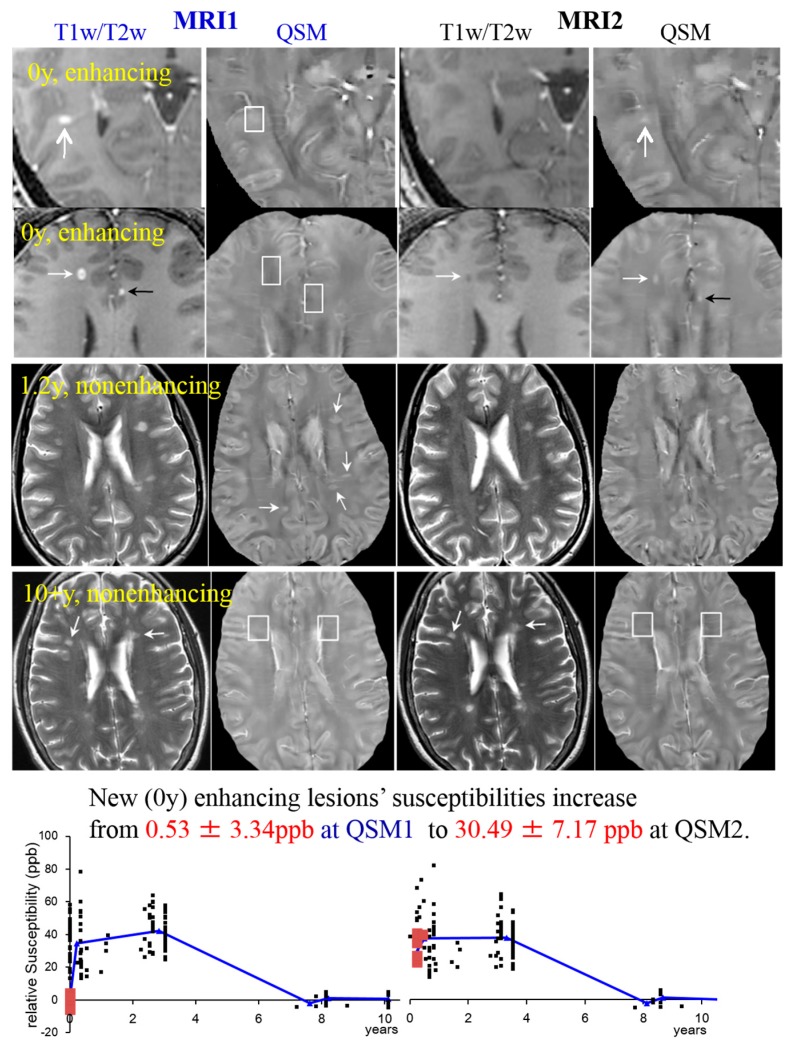
MS lesions on T1w (1st and 2nd row)/T2w (3rd and 4th row) and QSM at MRI1 (**left, in blue**) and a follow-up study at MRI2 (**right, in black**). The white boxes indicate the position of lesions identified on T1w, which are invisible on QSM. White arrows point at lesions in general; black arrows point at transient lesions, which disappear (appear T1 isointense) in the follow-up study (MRI2). Lower graphs: 32 cases are shown to exemplify lesions at various ages. The susceptibility of acute lesions (0 y) relative to NAWM jumped from MRI1 to MRI2 (red squares at bottom graph, interval between MR examinations = 0.43 ± 0.16 years). (Source: [42]).
- T2* weighted imaging (T2*w). Lesion magnetic susceptibility increases, as microglia degrade and remove diamagnetic myelin fragments [10,162,163], and as m/M take in highly paramagnetic iron [9,10,13,20,31]. Thus, in recent years, GRE sequences have been actively used to gain new insights into MS lesion inflammation activity [8,15,17,18,19,21,22,23,24,25,28,29,38,42,164,165,166]. Hypointense rings on T2*w images correlated with histologically demonstrated iron-laden microglia present at the edge of chronic active lesions [147], but both solid and shell lesions will generate rings on T2*w images [8,167] (shown in Figure 4).
- **Susceptibility Weighted Imaging (SWI)** SWI has been utilized to see differences of iron content in healthy subjects *vs.* MS patients [23] and to detect lesions in GM and WM [17,24,30,168,169].

- **T2-weighted imaging (T2w).** T2 relaxometry has been applied to detect iron accumulation in DGM of MS patients showing a shortening of the T2 relaxation time for MS patients [19]. However, it is currently not possible to attribute changes in relaxation times to specific pathologic changes, which is crucial for lesion characterization [160]. Furthermore, in contrast to WM lesions and leukocortical lesions, intracortical lesions remain largely undetected in T2 [161]. WM lesions appear hyperintense on T2 due to demyelination (shown in Figure 3 and Figure 6).

**Figure 4:**
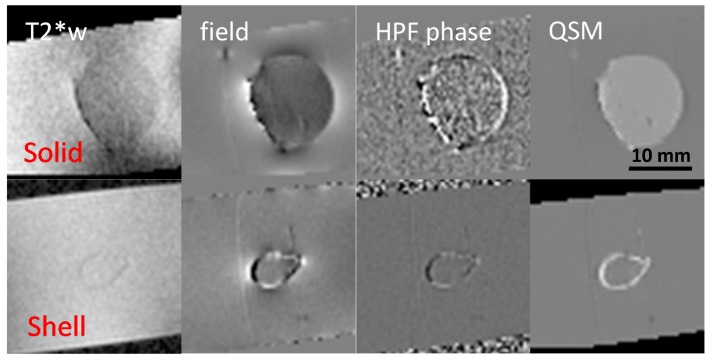
Solid and shell susceptibility (ultrasmall superparamagnetic iron oxide particles (USPIO)) object appearance in MRI techniques. Solid and shell lesions can be identified on QSM images; however, they both appear shell-like on phase images. (Source: [170]).

#### 5.2.2. Semi-Quantitative Iron Detection Using Conventional MRI Contrasts

Iron quantification is highly desirable for monitoring disease progression and therapeutic outcome as well as for enabling longitudinal or cross-center studies. Here, we summarize three semi-quantitative methods that have been used for measuring iron in MS:

- **Phase Imaging.** MS lesions may appear isointense or hypointense on phase images [24,166]. It should be noted that both solid and shell (ring in 2D sectional image) lesions in MS brains have shell appearance, according to physics law and experimental data [170] (shown in Figure 4). The difficulty in interpreting GRE phase data of MS [162,164,165,166] is because phase is not a local tissue property, but a weighted summation of the magnetic properties of the surrounding tissue. Thus, phase cannot be used to quantify local iron density, which was validated experimentally [66].
- R2* Mapping. An increased R2* has been observed in the DGM structures of MS patients, indicating increased iron content [21,35,171,172]. T2* or R2* mapping has been used to investigate intracortical and leukocortical lesions [147,173,174].
- **Magnetic Field Correlation Imaging (MFC)** MFC has been utilized to measure increased iron content in MS patients [15,34]. However, MFC has the most R2* limitations discussed above.

### 5.3. Quantitative Susceptibility Mapping (QSM)

Important for MS applications, QSM is more sensitive than R2* in detecting pathological changes in the basal ganglia [46], allowing iron quantification in completely demyelinated areas [170]. QSM quantitation enables time course investigation that is organizing MS lesion heterogeneity into lesion dynamics [42]. Correcting for myelin contribution using myelin water fraction (MWF) [175,176,177] and diffusion tensor imaging (DTI) [41,178], QSM can quantitatively map iron, possibly representing inflammatory microglia [41]. Here, we summarize QSM and pathology findings in MS.

#### 5.3.1. Shell *vs*. Solid Lesions [170]

Differentiating solid from shell lesions is important for understanding MS lesion pathology [9,10], but this is challenging on phase images. Solid and shell susceptibility sources were correctly reconstructed on QSM, while the high pass filtered (HPF) phase and field images depicted both geometries with the same shell-like pattern (Figure 4). Twenty MS cases were reviewed by a neuroradiologist; of the 21 lesions that appeared as rings on HPF phase, 14 (66%) appeared as solids and seven (33%) appeared as rings on QSM.

#### 5.3.2. MS Lesion Susceptibility Time Course Study [42]

Thirty-two clinically confirmed MS patients underwent two MR exams (denoted by MRI1 and MRI2) with 0.43 ± 0.16 year (y) interval on a 3T scanner. MRI included T2w, T1w, T1w+c and QSM (20, 21). Ages of 162 lesions were measured by examining their first appearance on prior MRIs (over past 0.3–10.6 y) in PACS. The susceptibilities relative to NAWM and temporal rates of change in lesion susceptibility relative to CSF were 0.53 ± 3.34 ppb (parts per billion) and 9.29 ± 1.84 ppb/month(m) for new enhancing lesions (age = 0 y), 38.0 ± 13.6 ppb (higher than NAWM, *p* < 0.01) and 1.3 ± 2.3 ppb/m (not different from zero, *p* = 0.38) for nonenhancing lesions at age = 0–4 y, and 4.67 ± 3.18 ppb and −0.11 ± 0.55 ppb/m for old nonenhancing lesions (age > 7 y). Examples are presented in Figure 3, showing that lesion susceptibility initially jumped from that of NAWM to high values for 0 y enhancing lesions (top two rows), became stable at high values for the 1.2 y nonenhancing lesions (3rd row), and decayed back to that of NAWM for the 10 + y nonenhancing lesions (4th row). Individual lesion values at MRI1 and MRI2 time points (Figure 3, bottom row), clearly demonstrate the initial jump (red points).

#### 5.3.3. QSM Is More Sensitive Than R2* in Detecting MS Basal Ganglia Change [46]

Sixty-eight patients (26 clinically isolated syndrome (CIS), 42 relapsing-remitting MS) and 23 healthy control (HC) subjects underwent 3T MRI. QSM and R2* maps were reconstructed from the same 3D multiecho spoiled GRE sequence. QSM successfully detected the difference in caudate nucleus and putamen between HC and CIS subjects, and difference in globus pallidus between HC and MS subjects while R2* failed to do so. Susceptibilities were higher with increasing neurologic deficits (*r* = 0.34, *p* < 0.01) (Figure 2).

#### 5.3.4. QSM, Laser Ablation Inductively Coupled Plasma Mass Spectrometry (LA-ICP-MS) and Immunohistochemical (IHC) Labeling for Myelin Basic Protein (MBP) and Myeloid Cells (CD68) [41]:

**Figure 5:**
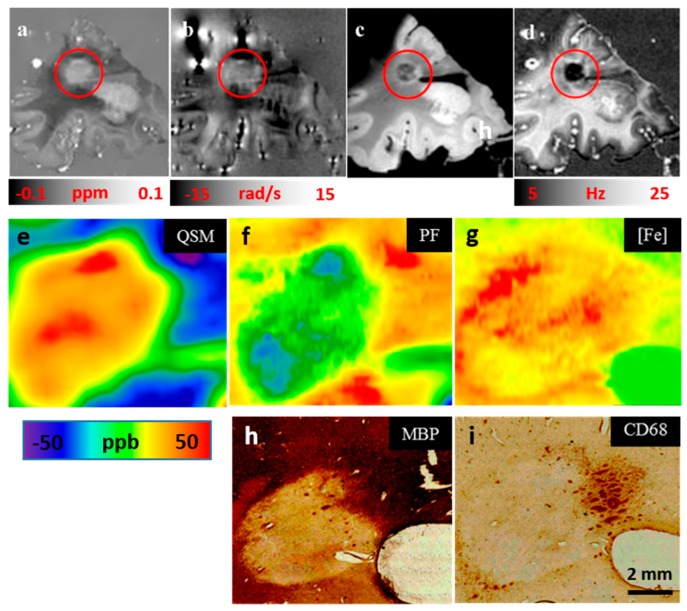
WM lesion (in red circle) appearing hyperintense on QSM (**a**); indicating high susceptibility and corresponding significant iron deposition, isointense on phase (**b**); hypointense on T2*w (**c**) and R2* (**d**). Magnification of lesion within red circle (**e-i**): The LA-ICP-MS measures all forms of iron (Fe^2+^, Fe^3+^), shown in (**g**); QSM mainly reflects ferritin-stored Fe^3+^ (**e**); Thus, iron maps were converted into susceptibility maps assuming a molar susceptibility of iron similar to that of ferritin at room temperature. Using the Langevin equation, iron contributes χ_[Fe]_ = 1.4 ppb*[Fe], where [Fe] represents the local iron concentration. The phospholipid map (PF) (**f**), representing myelin, was calculated as χ_Myelin_ = QSM − 1.4 ppb*[Fe] assuming that myelin is the major susceptibility component in WM [163]. PF (**f**) and MBP (**h**) indicate demyelination in the lesion center. CD68 stain (**i**) and iron map (**g**) indicate iron-rich microglia at the lesion rim. (Source: [41]).

Both iron deposition and demyelination in MS lesions can cause a susceptibility increase as measured by QSM [179], but their relative contributions are unclear. LA-ICP-MS [70] provides highly sensitive elemental analysis. IHC labeling for MBP and CD68 detects the presence or absence of myelin and myeloid cells, respectively. We performed MRI (QSM, phase, T2*w, R2*), LA-ICP-MS, MBP and CD 68 labeled ICH on a WM MS lesion (Figure 5). Quantitative [Fe] maps, acquired with LA-ICP-MS were converted to susceptibility 1.4 ppb*([Fe]/μg/mL) using the molar susceptibility of 3.78 Bohr magnetons per iron at room temperature [128] and a tissue density of 1.04 g/cm^3^ [180]. The myelin phospholipid fraction (PF) was estimated from the residual susceptibility QSM − 1.4*[Fe] using the mean molar susceptibility −449 ppb (L/mol). The lesion had a negligible PF (<0.07 at lesion center; ~0.2–4 for NAWM) and the interior showed susceptibility 29 ± 7.5 ppb on QSM, which is approximately fully explained by 27 ± 7.5 ppb contribution from [Fe]. The IHC stains (Figure 5: MBP, CD68) show absence of myelin and low level of m/M in the lesion center, indicating a possibly inactive lesion aged 1–3 y (Figure 5).

#### 5.3.5. Iron Presence in a White Matter Lesion Can Often Be Unequivocally Identified on QSM

**Figure 6:**
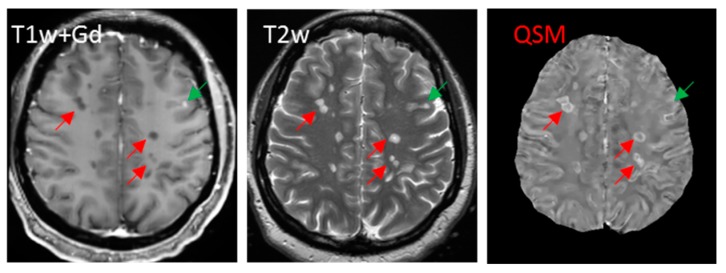
Non-enhancing lesions appear larger with hyperintense rim on QSM (red arrows) than on T2w, while enhancing lesions appear isointense on QSM (green arrow).

Iron in white matter lesions can be identified at the hyperintense rim on QSM, in the QSM lesion volume lying outside the lesion volume on T2-weighted imaging, or with the positive QSM value relative to the CSF [41,147,181,182] (Figure 6). The myelin contribution to susceptibility may be compensated using quantitative myelin water fraction imaging and diffusion tensor imaging, providing absolute iron quantification in general.

In summary, iron-sensitive imaging of multiple sclerosis offers the possibility to monitor several aspects of MS pathology, in particular chronic microglial activation and shifts in iron concentrations in NAWM that cannot be measured by conventional MRI methods.

## 6. Adding QSM in Clinical MS MRI Protocol to Improve Inflammation Monitoring

**Figure 7:**
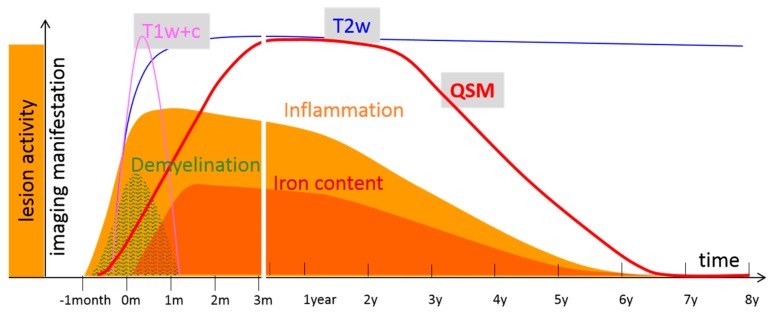
Lesion development over time showing various MS lesion activities (inflammation, iron deposition and emission (iron content)) and their manifestations on MRI (T1w+c, T2w, and QSM). Early inflammatory activity causes BBB damage, which is captured as Gd-enhancement on T1w+c; Immediate demyelination and iron deposition involving activated microglia cause rapid increase in magnetic susceptibility, which can be measured on QSM during MS lesion formation and development (approximately within the first 3 years (y)); Eventual old lesions (approx. >3 y) start to lose iron, which cancels the susceptibility increase of residual demyelination as measured by QSM. This time course indicates that QSM might be more sensitive than T1w+c in detecting inflammatory activity in lesions. QSM can detect both early and late inflammatory activity (remyelination is not frequently observed and not included here for simplicity).

Noninvasive MRI is critical for the diagnosis and surveillance of MS patients and is nowadays the main window for studying this neurodegenerative disease. Current inflammation assessment in clinical practice is based on gadolinium (Gd) enhancement on contrast enhanced T1-weighted (T1w+Gd) MRI [14]. However, Gd-enhancement is only an indirect measure of inflammation that is preceded and outlasted by the infiltration of immune cells [16] and is not reflective of the activation of resident innate immune cells of the central nervous system (CNS) [183]. T1w+Gd only offer a small time-frame of about three weeks for lesion detection, when the BBB is open for infiltration with immune cells [184]. Thus, T1w+Gd cannot capture the multiplicity of MS disease processes, resulting in poor pathological specificity [26] (Figure 7).

QSM can be used to alleviate this situation. Myeloid cells during the Gd-enhancing period phagocytose myelin fragments, a process that is reflected in the initial lack of change in the susceptibility of active lesions on QSM. After the BBB is reconstituted, myeloid cells metabolize diamagnetic myelin fragments, and at the same time or afterwards, microglial cells at the lesion periphery accumulate paramagnetic iron [10]. Both myelin debris removal and iron accumulation likely contribute to the increase in lesion susceptibility observed on QSM. MS lesions are hyperintense, typically with bright lesion rims on QSM for about four years [42], that can be interpreted as iron [41] (Figure 6). Therefore, including QSM in MRI protocols for MS patient imaging may provide more information about the inflammatory status of lesions than Gd-enhancement alone (Figure 7).

Using QSM and T2w together can allow for accurate identification of lesions in MS patients without Gd injection (Figure 6), utilizing the fact that the magnetic susceptibility of an MS lesion increases rapidly as it changes from Gd-enhancing to non-enhancing [42]. We performed a preliminary study with the following findings [185]: a total of 126 new lesions were evaluated in 52 patients. Eighty-three lesions were identified as Gd-enhancing with their susceptibility values (2.46 ± 6.40 parts-per-billion (ppb)) significantly lower than those of non-enhancing lesions (19.92 ± 7.80 ppb, *p* < 0.005). Receiver operating characteristic (ROC) analysis for discriminating enhancing and non-enhancing lesions using susceptibility values showed an area-under-the-curve of 0.956. A cutoff-value of 11.2 ppb for QSM-measured susceptibility provided a sensitivity of 89.2% and specificity of 90.7%. Therefore, QSM can be used in routine MRI monitoring of new MS lesion activity to accurately identify the BBB leakage status of T2w lesions without gadolinium injection, which would be useful particularly for MS patients with contraindications for Gd injection.

## 7. Conclusions

Iron homeostasis in the MS brain is profoundly perturbed, with iron-overload in lesional myeloid cells and in deep nuclei on one hand, and diminished iron concentrations in NAWM and chronic cortical lesions on the other. These shifts are likely to contribute to low-grade chronic inflammation, oxidative stress and neurodegeneration. QSM can be used to detect iron changes and may thus improve our ability to monitor therapeutic efficacy and progression in MS.

In clinical practice, iron-sensitive imaging could therefore be used to aid treatment decisions in patients that appear clinically and radiographically stable but may have iron-positive, activated microglia within lesions. Moreover, accelerated resolution of iron accumulation could become an additional measure for treatment efficacy to reduce low-grade inflammation in established lesions.
